# Complete Genome Sequence of Escherichia coli Siphophage Schulenberg

**DOI:** 10.1128/MRA.01053-19

**Published:** 2019-09-26

**Authors:** Madeline Rivera, Heather Newkirk, Russell Moreland, Mei Liu, Jolene Ramsey

**Affiliations:** a Center for Phage Technology, Texas A&M University, College Station, Texas, USA; b Department of Animal Science, Texas A&M University, College Station, Texas, USA; Queens College

## Abstract

Escherichia coli bacteria and their infecting bacteriophage exist within the gut. Here, we present the complete genome of Schulenberg, an E. coli siphophage similar to phages of the subfamily *Guernseyvirinae*. Schulenberg encodes 85 proteins, 33 of which have predicted functions.

## ANNOUNCEMENT

Escherichia coli is a Gram-negative facultative anaerobe commonly found in the lower gastrointestinal tract of mammals (1). Diversity within gut-dwelling bacterial populations aids in digestion and maintenance of healthy immune function. Some groups have proposed that phage therapy manipulating gut microbiota may prove useful in treating chronic diseases in humans (2). Here, we report the E. coli-infecting siphophage Schulenberg.

Bacteriophage Schulenberg was isolated on host E. coli 4s from a filtered (0.2-μm pore size) private septic system sample collected in Franklin, TX (3). Both phage and host were grown aerobically at 37°C in Luria broth (BD), and standard soft agar overlay methods were used (4). Phage morphology was determined using transmission electron microscopy at the Texas A&M Microscopy and Imaging Center after staining with 2% (wt/vol) uranyl acetate (5). Phage genomic DNA libraries were prepared after isolation with the shotgun library preparation modifications to the Promega Wizard DNA clean-up system using Illumina TruSeq Nano low-throughput kits and were sequenced with an Illumina MiSeq instrument with 250-bp paired-end reads using V2 500-cycle chemistry (6). The 4,413 raw sequence reads were quality controlled with FastQC (www.bioinformatics.babraham.ac.uk/projects/fastqc) and then trimmed using the FASTX-Toolkit (http://hannonlab.cshl.edu/fastx_toolkit/). Genomes were assembled with SPAdes v3.5.0 at default settings into a single contig of circular assembly at 7.1-fold coverage (7). The product was confirmed and closed using PCR (forward primer, 5′-GAGAAGTTACGAGAGTACAGGAGTAATA-3′; reverse primer, 5′-GCCAACACCTTCTCCATCT-3′) and Sanger sequencing of the product. Glimmer v3.0 and MetaGeneAnnotator v1.0 and ARAGORN v2.36 were used to call protein-coding and tRNA genes, respectively (8–10). Rho-independent termination sites were annotated from TransTermHP v2.09 (11). Gene functions were predicted using domain scans with InterProScan v5.33-72, TMHMM v2.0, and BLAST v2.2.31 at default parameters (with a 0.001 maximum expectation value) for the NCBI nonredundant and UniProtKB Swiss-Prot/TrEMBL databases (12–15). Structural similarity predictions were carried out with the HHSuite v3.0 HHpred tool (multiple-sequence alignment [MSA] generation with the HHblits ummiclus30_2018_08 database and modeling with PDB_mmCIF70) (16). Sequence similarity to other phages was calculated using progressiveMauve v2.4.0 (17). All tools used for annotations are available on the Center for Phage Technology Galaxy and Web Apollo instances (http://cpt.tamu.edu/galaxy-pub/) (18, 19).

The 44,748-bp double-stranded DNA genome of siphophage Schulenberg was predicted to be packaged by a headful mechanism using PhageTerm (20). Schulenberg has 85 predicted protein-coding genes, 33 of which have a predicted function, no identifiable tRNA genes, a coding density of 94.8%, and a G+C content of 49.9%. The most closely related phage to Schulenberg is *Escherichia* phage VB_EcoS-Golestan (GenBank accession number MG099933) within the subfamily *Guernseyvirinae*, with 67.63% nucleotide similarity and 58 similar proteins (21). Unlike other phages in the *Guernseyvirinae* subfamily, Schulenberg contains no detectable inteins, but it carries two freestanding HNH endonucleases (NCBI accession numbers QEG06793 and QEG06824). A superinfection immunity protein (NCBI accession number QEG06814) with similarity to the *Escherichia* phage T4 Imm protein (NCBI accession number NP_049660) was also found. The Schulenberg holin/antiholin pair (NCBI accession numbers QEG06859 and QEG06860) and endolysin (NCBI accession number QEG06861) are genetically separate from the partially overlapping i-spanin/o-spanin (NCBI accession numbers QEG06797 and QEG06798).

### Data availability.

The genome sequence and associated data for phage Schulenberg have been deposited under GenBank accession number MK931438, BioProject accession number PRJNA222858, SRA accession number SRR8892142, and BioSample accession number SAMN11408657.
